# Radiomics and Deep Learning Interplay for Predicting MGMT Methylation in Glioblastoma: The Crucial Role of Segmentation Quality

**DOI:** 10.3390/cancers17213417

**Published:** 2025-10-24

**Authors:** Francesca Lizzi, Sara Saponaro, Alessia Giuliano, Cinzia Talamonti, Leonardo Ubaldi, Alessandra Retico

**Affiliations:** 1 National Institute for Nuclear Physics, Pisa Division, 56127 Pisa, Italy; francesca.lizzi@pi.infn.it; 2 Candiolo Cancer Institute, FPO-IRCCS, 10060 Candiolo, Italy; sara.saponaro@ircc.it; 3 Department of Physics, University Hospital S. Chiara, 56126 Pisa, Italy; alessia.giuliano@ao-pisa.toscana.it; 4 National Institute for Nuclear Physics, Firenze Division, 50125 Firenze, Italy; cinzia.talamonti@unifi.it (C.T.); leonardo.ubaldi@unifi.it (L.U.); 5 Department Biomedical Experimental and Clinical Science “Mario Serio”, University of Firenze, 50134 Firenze, Italy

**Keywords:** glioblastoma, MRI, radiomic, CNN, segmentation

## Abstract

**Background/Objectives:** Glioblastoma (GBM) is the most malignant subtype of glioma and shows the poorest prognosis with a median survival time of 15 months. The methylation status of the Methylguanine-DNA Methyltransferase (MGMT) was proven to be a crucial factor in selecting the most appropriate therapy. Currently, it is assessed through brain biopsy, which is a highly invasive and very expensive technique. For these reasons, in recent years, the possibility of inferring this information from multi-parametric Magnetic Resonance Imaging (mpMRI) has been widely explored. However, substantial differences in performance are reported in the literature. **Methods:** In this study, we developed several models based on either radiomic or deep learning approaches and a mixture of them using mpMRI for the MGMT status assessment using the public dataset UPENN-GBM, available on The Cancer Imaging Archive. Despite the tests performed using all MRI acquisitions and different methodological approaches, we did not obtain sufficiently reliable performance to direct the therapeutic path of patients. We thus investigated the impact of segmentation quality on MGMT status prediction since the UPENN-GBM dataset contains both automatic and manual refined segmentation masks. **Results:** We found that performance obtained through radiomic features computed on manually segmented tumors was significantly higher compared to that obtained using automatic segmentation, even when the differences between segmentation masks, measured in terms of Dice Similarity Coefficient (DSC), is not significantly different. **Conclusion:** This could be the reason why very different MGMT classification performance is typically reported and suggests the creation of a benchmark dataset, with high-quality segmentation masks.

## Simple Summary

Glioblastoma (GBM) is the most malignant subtype of glioma and the methylation status of the Methylguanine-DNA Methyltransferase (MGMT) was proven to be a crucial factor to select the most appropriate therapy, which is currently assessed through brain biopsy. In this study, we investigate the possibility of inferring this information from multi-parametric Magnetic Resonance Imaging (mpMRI) through several models based on either radiomic or deep learning using the public dataset UPENN-GBM, available on The Cancer Imaging Archive. We did not obtain sufficiently reliable performance to direct the therapeutic path of patients. We thus investigated the impact of segmentation quality on MGMT status prediction since the UPENN-GBM dataset contains both automatic and manual refined segmentation masks, finding that performance is significantly dependent on the segmentation quality.

## 1. Introduction

Glioblastoma (GBM) is the most malignant and pervasive subtype of glioma and is the most prevalent kind of primary brain tumor in adults [1]. Despite some advances in standard multi-modal treatment, including surgical resection followed by adjuvant chemotherapy and radiotherapy, it has the poorest prognosis, with a median survival time of 15 months [2]. The identification of O(6)-Methylguanine-DNA Methyltransferase (MGMT) methylation status has been proven to be fundamental for selecting the most accurate treatment strategy and improve success rates for GBM treatment [3]. The reference standard for evaluating the MGMT methylation status is the analysis of biopsy samples using expensive and complex molecular techniques [4]. For this reason, the scientific community is interested in focusing on non-invasive techniques based on proteomics and protein expression [5], as well as identifying links between genetic characteristics and medical imaging features based on deep learning and radiomics.

Some studies have developed AI and radiomics-based tools to predict the MGMT methylation status of GBM from MRI [6,7]. It is interesting to notice that there are two clear trends in the literature: the first found no or poor correlation between mpMRI and MGMT status, while in the second trend, promising and high performances are found by previous studies. For example, Han et al. [8] developed a recurrent neural network that achieves an AUC equal to 0.61 on their test set (without reporting a confidence interval). Lu et al. [9] developed several machine and deep learning models on radiomic and clinical features and reported an accuracy that spans from 45% to 60% on the MGMT prediction. Other studies showed poor performances, especially when passing to an unseen independent test set using radiomic features and deep learning algorithms [10,11]. Finally, Saeed et al. [12] reported no correlation at all between MRI and MGMT promoter methylation status. On the other hand, some studies reported very promising results. For example, Do et al. [2] implemented a genetic algorithm for the feature selection, reaching an accuracy equal to 92.5% on a 5-fold cross-validation. Koska et al. [13] claim an AUC equal to 0.90 in the MGMT classification.

In this study, we implemented several pipelines with the aim of getting the most out of the information contained in multi-parametric MRI imaging (mpMRI) in order to predict the MGMT promoter methylation status for patients with GBM included in a TCIA publicly available dataset. We implemented both single-modality and multi-modal approaches using both radiomic features, deep learning, and a mixture of them in order to investigate whether it is possible to reliably predict the MGMT promoter status from imaging data. The first key point of this work is the use of a robust validation methodology for both deep learning and radiomic pipelines, consisting of a stratified 5-fold cross-validation along with a separate balanced test set. Our results align well with that part of the literature stating that there is no correlation between MGMT methylation status and mpMRI [7]. To strenghten our contribution, we explored additional insights of the used dataset, particularly on how the tumor segmentation impacts the robustness of MRI radiomic features and their predictive power. Given that the dataset includes both automatic and manually refined segmentation masks, we were able to achieve this goal in a reproducible way.

## 2. Materials and Methods

### 2.1. Data

The data used in this study was the Upenn-GBM dataset [14], a dataset of multi-parametric MRI scans of patients with de novo glioblastoma that has been made publicly available on The Cancer Imaging Archive (TCIA) (https://www.cancerimagingarchive.net/ (accessed on 1 September 2024)) [15]. The UPenn-GBM collection includes mpMRI scans consistently acquired at the University of Pennsylvania Health System (UPHS), along with clinical and demographic data, as well as molecular status information, including information on methylation of the MGMT promoter based on pyrosequencing.

**Figure 1:**
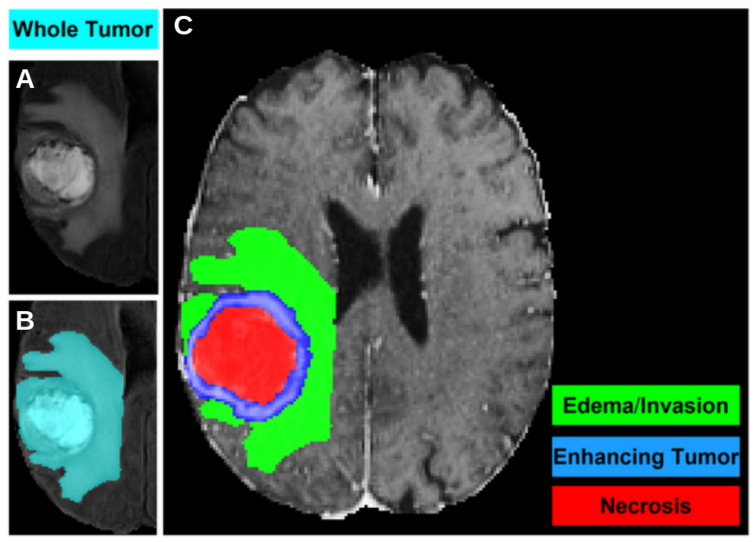
An axial slice of a T1-weighted MRI of the dataset, where the different segmentation masks are visible: (**A**) the tumor, including all sub-portions, is shown in the original image grayscale; (**B**) the Whole Tumor mask, i.e., the union of the three previous masks, is shown in cyan; (**C**) the necrosis area is shown in red, the enhanced part of the tumor in blue, the edema in green.

All images were pre-processed using a standardized protocol that included the coregistration of all mpMRI scans to a common anatomical atlas, resampling to an isotropic resolution of 1 ${\mathrm{mm}}^{3}$, and skull-stripping. Each patient had pre-operative images in four modalities (T1, T1-contrast enhanced, T2, and FLAIR) along with Diffusion Tensor Images (DTI), including the Fractional Anisotropy (FA) and the Axial Diffusivity (AD) maps that are exploited in this study. Two different types of segmentations are available. The first segmentation was performed using the STAPLE label fusion technique [14], which combines the results of three top-ranked deep learning algorithms from the BraTS challenge: DeepMedic, DeepSCAN, and nnUNet. The second segmentation is a manual revision and correction of the first, conducted by expert clinicians for a subset of 232 subjects. The output of the segmentation delineates different masks, highlighting various parts of the tumor: the enhancing part of the tumor core (ET), the non-enhancing part of the tumor core (NET), and the peritumoral edema (ED). These are illustrated for a representative subject of the dataset in Figure 1.

The number of subjects for which both segmentation masks and the MGMT promoter methylation status are available is limited to 258 subjects (109 MGMT+ and 149 MGMT−). Finally, with regard to the insights on the radiomic features robustness and the dependence of results from the segmentation, we used only the subjects that have the manually refined segmentations, of which there are 55 (20 MGMT+ and 35 MGMT−).

### 2.2. Analysis Flow

Since the number of MRI sequences associated with each patient is too high to be analyzed as a set of 3D volumes, we set up the analysis in the following way:

- The first step was the identification of structural and diffusion MRI sequences that contain a relevant amount of information related to the classification of MGMT promoter methylation status. To achieve this goal, the T1, T2, T1Gd, and FLAIR sequences were analyzed using both a radiomic and a deep learning approach, while DTI images have been used only to train and evaluate the radiomic approach.
- Then, we developed different joint models using the Fractional Anisotropy (FA) and the Axial Diffusivity (AD) maps to train a 3D Convolutional Neural Newtork (CNN) to explore whether these volumes contain information about the MGMT methylation status.
- Furthermore, we trained a multi-modal 3D CNN along with radiomic features in a joint fusion approach to maximize the performance of the classifier.
- Finally, since the performance we obtained was not satisfactory to claim for a reliable prediction of the MGMT promoter status, we end our analysis with specific insights into the dependence of the robustness and the predictive power of radiomic features on the quality of provided segmentation masks.

### 2.3. Mri Data Pre-Processing

Before proceeding with the several analysis approaches enumerated above, structural MRI images were pre-processed according to the procedure presented by Ubaldi et al. [16]. The procedure starts with an intensity normalization step to make the gray value distributions of images acquired with the same MRI sequence across different subjects comparable. The intensity value of each voxel was transformed by subtracting the median value and dividing by the *IQR* of the intensity values of the brainstem, using the formula:

$$x_{i}^{Norm\_Brainstem}=\frac{x_{i}-\mathrm{median}\left({\bar{x}}_{Brainstem}\right)}{IQR}.$$

The segmentation of the brainstem is a prerequisite to implementing this normalization strategy. For this purpose, the mpMR images of each patient and an atlas including the brainstem mask were coregistered to the MNI space using ANTsPy. The SyNRA transformation in ANTsPy, which includes a rigid, an affine, and a deformable transformation with mutual information as the optimization metric, was applied. After coregistration, a check to ensure the brainstem mask does not intersect the tumor mask was carried out; then, all images were intensity-normalized based on the Norm_Brainstem procedure. The intensity-normalized MRI images are the input data to all radiomic and deep learning approaches presented in this paper.

### 2.4. Computation of Radiomic Features

The computation of the radiomic features was performed using the open-source Python package *PyRadiomics* (v3.0.1) (https://pyradiomics.readthedocs.io/ (accessed on 1 September 2024)) [17]. This platform was validated by the developers against the IBSI benchmark values [18]. The radiomic features were computed for all available MRI sequences for each patient, utilizing the NET, ED, ET, and the Whole Tumor (WT) masks derived from both segmentation methods. For each region, we extracted 107 features which included the following:

- 18 histogram-based features (also known as First Order Statistics or intensity features) computed on pixel gray-level histograms;
- 14 shape-based features, dependent only on the shape of the mask;
- 75 texture-based features, derived from the gray-level co-occurrence matrix (GLCM), gray-level size zone matrix (GLSZM), gray-level dependence matrix (GLDM), gray-level run length matrix (GLRLM), and neighboring gray tone difference matrix (NGTDM).

### 2.5. Selection of More Informative Structural MRI Sequences

As mentioned in Section 2.2, the first part of the analysis is focused on understanding which structural MRI sequence may be used to predict the target with the aim of optimizing the computational cost. To this purpose, we trained a Random Forest (RF) algorithm according to a stratified 5-fold cross-validation scheme on the radiomic features extracted from all the available structural sequences (T1, T1Gd, T2, and FLAIR) and using all the segmentation masks available (ET, ED, NET, and WT, which is the logical union of the previous three). The RF training process consists of training a number of decision trees on randomly selected data samples, obtaining a prediction from each tree, and then selecting the best solution by means of voting [19]. We used the *Random Forest Classifier* implemented in the *Scikit-learn* (https://scikit-learn.org/ (accessed on 1 September 2024)) [20] open-source machine learning Python library. We set the number of trees to the default value of 100 and the number of candidate predictors considered in each split to $\sqrt{n_{P}}$, where $n_{P}$ is the number of predictors.

**Figure 2:**
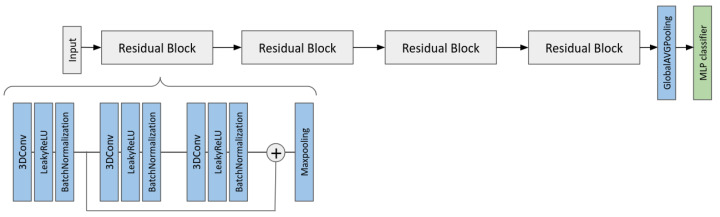
Architecture of a CNN designed to predict the methylation status of MGMT promoter from a single 3D MRI volume. The structure of a residual block is shown only for the first block and it is the same for the others. The number of convolutional filters increases from 16 to 64 for each block.

Moreover, we optimized, trained, and evaluated a 3D CNN for the prediction of the MGMT promoter methylation status using each single structural sequence without segmentation and masked with the WT segmentation. In Figure 2, a scheme of the CNN is reported. The training was performed according to a stratified 5-fold cross-validation scheme for 100 epochs with batch size equal to 2. The optimizer adopted was the Root Mean Square Propagation (*rmsprop*), the loss function was the binary cross-entropy, and the metric for the evaluation of the performance during the training was the accuracy.

For both RF and CNN, the metric we used to evaluate the classification performance was the Area Under the ROC Curve (AUC) [21]. To compare models, the same independent test set, containing 15 methylated and 15 unmethylated cases, was used.

### 2.6. Multi-Input CNN Models to Predict the MGMT Status

CNNs have proven to be one of the most powerful instruments to analyze images, offering both the possibility of exploiting the automated feature learning and their flexibility in being used to merge data of different sources [22]. However, as the amount of information available in this dataset of mpMRI data is too huge to allow the analysis of all multi-parametric data with a single DL model, we implemented a strategy to retain all the significant information while maintaining the computational burden acceptable for the available hardware, as detailed in the previous section. Moreover, we also trained a multi-branch CNN that takes as input both the FA and AD 3D maps. We proceeded using different models with the aim of merging the information extracted from the MRI sequences selected by the previous step of the analysis:

1. A multi-branch CNN that takes as input the two MRI volumes;
2. A multi-modal CNN-MLP that takes as input two MRI volumes and all the radiomic features.

**Figure 3:**
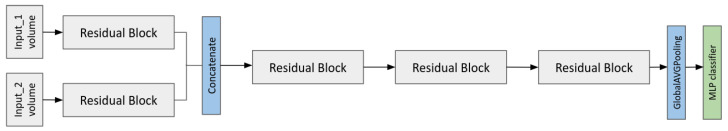
First joint model: the architecture allows the input of two 3D volumes that are processed by one residual block. Then, the activation maps computed after the first block are concatenated together in one tensor. The residual blocks are identical to the ones in Figure 2.

**Figure 4:**
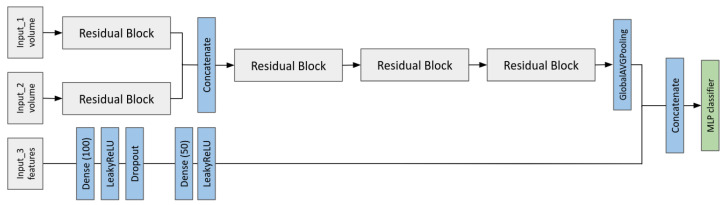
Second joint model: this architecture is made to take three different inputs. The upper part is identical to the one in Figure 3, while the lower branch is debuted to take radiomic features as input. The branches are then merged when the convolutional part of the net goes through a Global Average Pooling (GAP) and the resulting vector is concatenated with the output of the radiomic branch.

We have thus developed two main architectures that have been optimized with a random search: the first one is a multi-branch 3D CNN based on ResNet that is able to take as input two different volumes and the second one is a multi-modal 3D CNN with three branches, two for 3D images and one for the radiomic features. In Figure 3, the scheme of the first joint fusion model is reported, while in Figure 4, the architecture able to process both image volumes and radiomic feature is shown.

Both architectures have been trained with Adam as the optimizer, binary cross-entropy as the loss function, and accuracy as the metric during training. The training phases have been performed with a batch size equal to 2 for 150 epochs. The best epoch for each trained model has been selected using the validation set.

In any of the above cases, the same independent test set, containing 15 methylated and 15 unmethylated cases, has been taken apart in order to evaluate the performance of the classifier and to correctly compare the different models. Since the number of samples is limited, the training of each model has been performed with a stratified 5-fold cross-validation to maintain the classes balanced.

The metrics computed on the test set is the AUC while the reported error is the standard deviation of the models resulting from the 5-fold cross-validation.

### 2.7. Robustness of Radiomic Features with Respect to Variations in Segmentation Masks

In order to deepen our analysis, we evaluated the robustness of radiomic features when they are computed on different segmentation masks of the same tumor. For this reason, we evaluated the similarity between automatic and manual segmentation masks provided within the dataset. Subsequently, a RF algorithm has been trained with the features extracted on the two segmentation masks separately for the whole available dataset. Dice Similarity Coefficient (DSC) was used to compare automatic and manual segmentation, then the intraclass correlation coefficient (ICC) was evaluated for each feature between segmentation with the open-source Python package *Pingouin* (https://pingouin-stats.org/ (accessed on 1 September 2024)) [23]. The radiomic features were stratified based on their degree of robustness: poor (ICC ≤ 0.5), moderate (0.5 < CC ≤ 0.75), good (0.75 < ICC ≤ 0.9), and excellent (ICC > 0.9) robustness.

## 3. Results

### 3.1. Analysis to Select the More Informative MRI Sequences

As described in Section 2.5, first, we report the results in terms of AUC of the training of the RF classification made using all the different available segmentation masks (ED, ET, NET, and WT) on each of the structural MRI available sequences (T1, T1Gd, T2, and FLAIR) in Table 1.

It is apparent in the table that most of the models did not exceed the chance-level performance. Among the combinations of sequences and tumor areas that provided results above the chance level, the best result is obtained on the enhanced tumor mask (EN) of the T2 volume. However, taking into account the statistical error associated with this measure, which is ±0.05, the performance completely ovelaps the performances of most of the other combinations.

In addition to structural MRI, we also evaluated the classification performance on DTI-derived maps (AD, FA, RD, TR), as reported in Table 2. Also, in this case, the overall performance remains limited.

We report in Table 3 the classification results obtained with the 3D CNN models trained on 3D data of each MRI sequence separately. In both cases, the WT mask is applied and without segmentation. It is apparent in Table 3 that, even in this case, most of the models did not exceed the chance-level performance.

### 3.2. MGMT Status Prediction by Multi-Input CNN Models

In this section, we report the results of the two multi-input CNN models described above. The first model has been trained with the DTI images, in particular FA and AD and a second multi-input CNN, reported in Figure 4 that takes as input FA, AD and all the radiomic features computed on all the structural sequences. The use of FA and AD was chosen since we did not obtain promising results with the 3D CNNs as can be seen in Table 3. Training time went from two days for the first joint model to five days for the most complicated architecture (in Figure 4) on a Nvidia V100 with 32 GB of VRAM GPU. The results are reported in Table 4. Results show that while the combination of FA and AD reaches a performance that is slightly above the chance level, once we introduce the radiomic features too, the performance significantly decreases.

### 3.3. Dependence of the Classification Performance on Segmentation Quality

**Figure 5:**
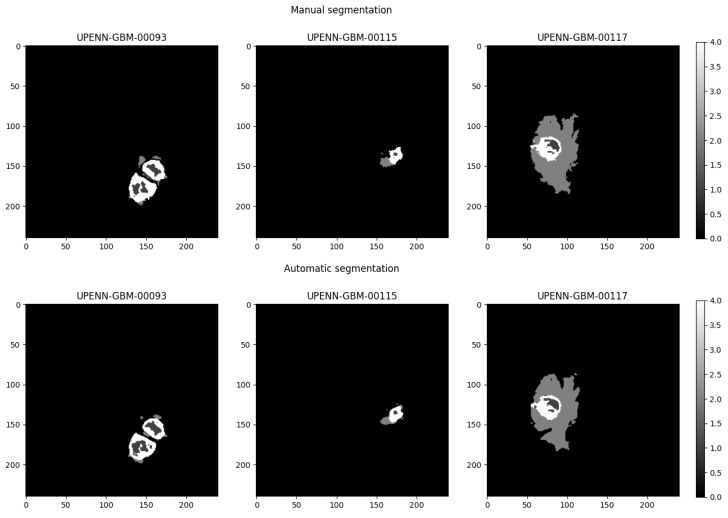
A comparison among the manual and automatic segmentation masks for three representative subjects.

Since the results we obtained did not highlight the possibility of predicting MGMT promoter methylation status reliably, and results slightly higher than the chance level were instead obtained in most cases by CNN models trained on single modalities without the application of segmentation masks, we decided to investigate the possible effect on the classification due to segmentation quality. We evaluated the two different sets of segmentation masks provided with the dataset, i.e., the manual and the automatic one. Figure 5 shows the tumor masks for the two different segmentation methods for three different representative subjects.

The evaluation of the overlap between the two sets of segmentation masks resulted in the mean and standard deviation DSC reported in Table 5. Dice scores vary across tumor regions, with highest values for whole tumor segmentation and lowest for edema and enhancing tumor parts.

**Figure 6:**
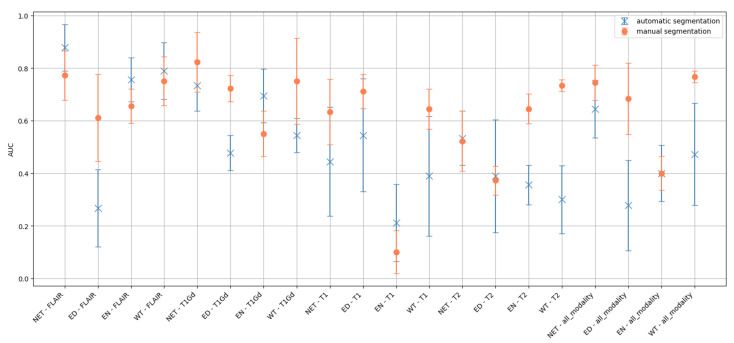
AUC of RF models trained on all the possible combinations of MRI sequences and segmentation areas to compare the classification performance of radiomic features computed with either manual or automatic segmentation.

We trained and evaluated a RF classifier, restricting the analysis to those patients with both manual and automatic segmentation. It is worth noting that the dataset used in this training is small since the manual segmentation masks are available for only 55 patients. This is also the reason why we could not use deep learning to evaluate the effect. Given the small number of training samples, we used only intensity and texture features. Figure 6 reports the AUC of models trained on all the possible combinations of MRI sequences and segmentation areas, evaluated on a test set obtained by randomly partitioning 15% of the data.

In the majority of combinations (12/20) (zone-modality), performance improves when using the mask reviewed by the clinician. To evaluate the statistical significance of this difference, we performed a paired *t*-test between the AUC values obtained for all modalities and all tumor zones with manual and automatic segmentation. The average values of AUC achieved with manual segmentation (AUC = 0.64±0.17) was significantly higher (*p*-value < 0.01) with respect to the average values obtained with automatic segmentation (AUC = 0.53±0.19), which settles at the chance level. Considering the specific case of the whole tumor area, the methylation status predictive power achieved with manual segmentation (AUC = 0.77±0.02) outperformed (*p* < 0.03) the one obtained with automatic segmentation (AUC = 0.47±0.20), which remains at the chance level. This represents the best performance obtained in this study, highlighting the role of manual segmentation.

## 4. Discussion

In this work, we presented several models trained on the public UPenn GBM dataset of multi-parametric MRI, available on The Cancer Imaging Archive [15]. We proceeded through the integration of different MRI sequences for the prediction of the MGMT promoter methylation status, developing several models based on either radiomic features analyzed with a RF classifier or deep learning-based approaches, including joint fusion models, using different tumor areas. We achieved poor performance with almost all the trained models, which is in line with some studies in the literature [8,9,10,11,12]. It is interesting to underline that the method we implemented in this paper is different from those cited above, since we implemented both radiomic and deep learning models, as well as a mixture of both with multiple MRI sequences in a stratified 5-fold cross-validation scheme along with a separate balanced test set. Interestingly, the method we developed is similar to the one developed by Koska et al. [13], who reported a very high correlation between MRI scans of the brain and the possibility to predict the MGMT promoter status. The main difference compared to their work is the robust statistical validation we used: specifically, Koska et al. used only a separate test set in a simple scheme including a train, a validation, and a test set. However, in small datasets like the one they used (577 samples), the possibility of having a lucky choice for the test set is likely. As underlined also by Doniselli et al. [6], the use of a strong statistical validation scheme tends to reduce the performance in this kind of study. To further deepen our analysis, we focused on the investigation of the possible effect of the segmentation of the tumor and of its sub-areas on the classification results, which might explain why the literature is so divided on this kind of problem. The model that achieved the best AUC (0.77±0.02) is the RF trained on the subset with manual segmentation. It is interesting to note that this model was trained only on 55 patients and, considering the WT segmentation, the difference between models trained with manual and automatic segmentations is significant. Finally, it is also interesting to note that the whole tumor segmentation shows the best agreement between manual and automatic segmentation according to the DSC score, as reported in Table 5. The variability introduced by such a small number of voxels can be the reason why such a high variability in the performance is generally reported in the literature on this classification problem. Moreover, another trend we found is that performances significantly decreased when passing from the evaluation on the validation sets of the cross-validation and an independent test set, showing the generally high amount of overfitting.

Our work aligns well with that part of the literature that found poor or no correlation between MGMT promoter methylation status and multi-parametric MRI data. This work presents some limitation: To correctly compare traditional and DL models, we did not perform any data augmentation. This leads to a relatively small number of samples in the training set that may not be sufficient to capture all the characteristics of patients with GBM.

## 5. Conclusions

In this work, we present an analysis on the possibility of predicting MGMT promoter methylation status using radiomic, machine, and deep learning applied to mpMRI. The best performance achieved is equal to 0.77±0.02 in terms of AUC and was obtained on a subsample of the dataset where manual segmentation was available. According to us, this result is not sufficient to address therapeutic paths of patients with GBM.

## Figures and Tables

**Table 1:** Mean AUC and Standard Deviation (STD) for each MRI sequence and tumor area (NET, ED, EN, WT) obtained with a Random Forest classifier trained on radiomic features. Legend for tumor areas: ED, edema; EN, enhancing tumor; NET, necrosis; WT, whole tumor.

MRI Sequence	Area	AUC ± STD
FLAIR	NET	** 0.50±0.08 **
	ED	0.40±0.04
	EN	0.52±0.04
	WT	0.39±0.07
T1Gd	NET	0.50±0.09
	ED	** 0.61±0.07 **
	EN	0.50±0.06
	WT	0.43±0.03
T1	NET	0.51±0.07
	ED	0.60±0.02
	EN	0.53±0.02
	WT	** 0.62±0.05 **
T2	NET	0.46±0.01
	ED	0.48±0.07
	EN	** 0.64±0.07 **
	WT	0.56±0.04
all_modality	NET	0.51±0.05
	ED	0.55±0.09
	EN	0.54±0.06
	WT	0.49±0.09

**Table 2:** Mean AUC and Standard Deviation (STD) for each DTI sequence and area (NET, ED, EN, WT) obtained with a Random Forest classifier trained on radiomic features. Legend for tumor areas: ED, edema; EN, enhancing tumor; NET, necrosis; WT, whole tumor.

DTI Sequence	Area	AUC ± STD
AD	NET	0.58±0.05
	ED	0.42±0.04
	EN	0.62±0.02
	WT	0.37±0.06
FA	NET	0.47±0.03
	ED	0.30±0.04
	EN	0.64±0.04
	WT	0.32±0.04
RD	NET	0.58±0.03
	ED	0.44±0.03
	EN	0.63±0.02
	WT	0.40±0.06
TR	NET	0.58±0.02
	ED	0.45±0.02
	EN	0.63±0.02
	WT	0.42±0.06
all_modality	NET	0.54±0.02
	ED	0.34±0.05
	EN	0.63±0.03
	WT	0.35±0.07

**Table 3:** Mean AUC and Standard Deviation obtained by the 3D CNN models on each MRI volume, with and without whole tumor (WT) segmentation mask.

MRI Sequence	Area	AUC ± STD over the Folds
T1	Whole Tumor	0.49±0.05
	No segmentation	0.68±0.12
T2	Whole Tumor	0.55±0.08
	No segmentation	0.60±0.09
T1Gd	Whole Tumor	0.44±0.05
	No segmentation	0.60±0.13
FLAIR	Whole Tumor	0.47±0.06
	No segmentation	0.41±0.07

**Table 4:** Mean AUC and its Standard Deviation (STD) obtained by the joint fusion multi-input CNN models on a combination of the FA and the AD 3D volumes, with and without the radiomic features extracted by the other MRI modalities.

Input Data	AUC ± STD over the Folds
Multi-input CNN with FA and AD	0.63±0.09
Multi-input CNN + MLP with FA,	0.56±0.12
AD and radiomic features	

**Table 5:** Mean DSC and Standard Deviation of the values resulting from the comparison of the two different segmentation methods, manual and automatic, on the different tumor areas.

Tumor Region	Dice Score
Whole Tumor	0.94±0.04
Necrosis	0.77±0.20
Edema	0.44±0.04
Enhancing Tumor	0.22±0.04

## Data Availability

No new data were created in this study. Data are publicly available at The Cancer Imaging Archive (https://www.cancerimagingarchive.net/ (accessed on 20 October 2025)).
